# Mycochemical Screening and Analysis, Antioxidant Activity, and Biochemical Composition of Fermentation Strain Snef1216 (*Penicillium chrysogenum*)

**DOI:** 10.1155/2020/3073906

**Published:** 2020-03-30

**Authors:** Aatika Sikandar, Mengyue Zhang, Yuanyuan Wang, Xiaofeng Zhu, Xiaoyu Liu, Haiyan Fan, Yuanhu Xuan, Lijie Chen, Yuxi Duan

**Affiliations:** ^1^Nematology Institute of Northern China, Shenyang Agricultural University, Shenyang 110866, Liaoning, China; ^2^College of Biosciences and Biotechnology, Shenyang Agricultural University, Shenyang 110866, Liaoning, China; ^3^College of Science, Shenyang Agricultural University, Shenyang 110866, Liaoning, China; ^4^College of Plant Protection, Shenyang Agricultural University, Shenyang 110866, Liaoning, China

## Abstract

Antioxidants are the radical scavengers that inhibit peroxidation and other free-radical processes, which in return safeguard different organisms from various diseases attributed to radical reactions. Synthetic antioxidants inhibit free radicals, but they also have harmful side effects. However, mycochemicals of natural fungal origin are safe and best substitutes for harmful synthetic chemical antioxidants. The prime objectives of the study include appropriate qualitative and quantitative mycochemical screening, antioxidant potential, and chemical composition of Snef1216 (*Penicillium chrysogenum*). The study has used aluminium chloride colourimetric method, Folin–Ciocalteu reagent assay, and DPPH (1,1-diphenyl-1-picrylhydrazyl) for analysis of total flavonoid content and phenol content and antioxidant activity, respectively. However, the presence of biologically active compounds was screened through gas chromatography-mass spectrometry (GC-MS). Quantitative analysis demonstrated the existence of flavonoids, glycosides, flavones, saponins, phenols, and catecholic tannins excluding alkaloids, terpenoids, steroids, and gallic tannins. The outcomes exposed total flavonoid content and phenolic content in *P. chrysogenum* were 85.31 ± 1.23 mg·QE/g and 135.77 ± 1.14 mg·GAE/g, respectively. Snef1216 (*P. chrysogenum*) displayed the highest free-radical scavenging activity with 63.86% inhibition of DPPH. The analysis confirms that Snef1216 (*P. chrysogenum*) is an alternative source of natural antioxidants. The obtained data have provided the foundation for its use in agricultural, environmental, and pharmaceutical industries.

## 1. Introduction

Free radicals are associated with numerous chronic and acute diseases in human beings such as asthma, atherosclerosis cataracts, diabetes, liver injury, and neurodegenerative disorder [1]. Antioxidants are useful constituents that are responsible for the inhibition of free radicals by abolishing their target site [2]. They have the capability to capture free radicals such as hydroperoxide and peroxide which constrain oxidation and cause degenerative arrays [3]. Synthetic antioxidants inhibit free radicals and also are have harmful side effects [4]. However, natural resources of mycochemicals are safe and better substitutes for chemical antioxidants [5]. Fungi are an important source of natural antioxidants because of their natural ability to produce secondary metabolites [6]. They contain antioxidants in the form of steroids, quinones, alkaloids, phenylpropanoids, tocopherol, phenols, tannins, lactones, terpenoids, triterpenes carotenoids, and flavonoids [7].

Essential mycochemicals contained in fungi such as phenolic contents have attained more interest due to their characteristics to inhibit disease by their antioxidant activity. Phenols and flavonoids are the major secondary metabolites of fungi [6]. Moreover, phenolic and flavonoids contents of fungal origin act as potential therapeutic agents, i.e., antimutagenic, antibiotics, anticarcinogenic, and antioxidants [8]. Fungi belonging to genera *Aspergillus*, *Chaetomium*, *Creosphaeria*, *Fusarium*, *Mucor*, *Penicillium*, *Tolypoclaidium*, and *Xylariaceae* showed extraordinary antioxidant potential [9].

Fungi belonging to the genus *Penicillium* are very common in nature [10]. They are filamentous fungi saprophytic organisms but may also colonize a wide range of materials [11]. They have an important role in bioremediation procedures by decomposition of various xenobiotics [12]. They produce a diversity of primary and secondary metabolites that are mainly owing to the existence of bioactive components such as flavonoids, alkaloids, minerals, proteins, phenols, tannins, vitamins, and antioxidants activities [5].

*Penicillium chrysogenum* is a common mold and present in almost all ecosystems [13]. Penicillin was the first antibiotic that was isolated from this species, and their biologically active secondary metabolites were the topic of intensive study in recent decades [11]. Many biologically active substances were isolated from *P. chrysogenum* such as tannins, alkaloids, terpenoids, and tannins [6]. It was found that the species of *Penicillium* produced scavenged DPPH radicals [14]. Penicillenols, secalonic acid D, and atrovenetin were isolated from *Penicillium* sp. as a potential antioxidant [15].

Strain Snef1216 (*Penicillium chrysogenum*) was identified by Jiang et al. [16]. Yao et al. [17] and Sikandar et al. [18] reported its efficiency against *Meloidogyne incognita* (root-knot nematode) in tomato and cucumber, respectively. However, mycochemical analysis and the presence of biologically active compounds have not been properly studied. Therefore, the current study was planned to appraise the presence of mycoconstituents, antioxidant activity, and chemical composition of fermentation strain Snef1216 (*Penicillium chrysogenum*) at the Nematology Institute of Northern China (NINC), Shenyang Agricultural University, Shenyang, Liaoning, People's Republic of China, during the year 2019.

## 2. Materials and Methods

### 2.1. Activation of Strain Snef1216 (*P. chrysogenum*) and Preparation of Fermentation

Fungal strain Snef1216 (*P. chrysogenum*) was obtained from the China General Microbiological Culture Collection Centre and kept at −80°C. To confirm the purity and activation of the strain, a small amount of the strain was added into PDA- (potato dextrose agar-) filled cavities and then kept in an incubator for 7 days at 25–28°C. The composition of nutritious medium for fermentation: 50 g sucrose, 8 g sodium nitrate, 2 g dipotassium phosphate, 0.4 g potassium chloride, 0.08 g magnesium sulfate heptahydrate, and 0.003 g ferrous sulfate heptahydrate were mixed in 1000 ml distilled water. The consequent mixture was boiled for 5-6 min and then poured 100 ml of medium into a 250 ml flask. The medium was sterilized in a steam autoclave machine (Zealway (Xiamen) Instrument Co., Ltd., Model no. GI54DS) for 30 min at 121°C, and five fungus cakes (approximately 1-millimeter diameter each) were added into the 100 ml medium. Then, the resulting mixture was placed in a shaker for 3 days at 150 rpm and 28°C. After 3 days, 100 ml of the new nutritious medium was added into each flask and then placed in a shaker for 8 days at 150 rpm and 28°C. Fermentation was filtered and stored at 4°C until use [16–18].

### 2.2. Qualitative Mycochemical Analysis

Qualitative analysis was done to assess the existence of biochemicals in the fermentation strain Snef1216 (*P. chrysogenum*) by using standard methods.

#### 2.2.1. Glycosides Test

Glycosides' existence was assessed by pouring 3 ml of 5% ferric chloride (FeCl_3_) and 3 ml distilled water into a 3 ml fermentation solution. After that, the mixture was heated for 15 min in a water bath. After cooling, 1.5 ml benzene was poured and shaken vigorously for 15 sec. The subsequent mixture was allowed to settle down for 1 min at room temperature. Addition of 5-6 drops of concentrated ammonia (NH_3_) into the mixture resulted in pink colour appearance confirmed the presence of glycosides [19].

#### 2.2.2. Alkaloids Test

Alkaloids were determined by the addition of 3 ml of 2% hydrochloric acid (HCl) into the dry fermentation, and the mixture was heated at 100°C for 15 min in a water bath. The filtrated mixture was equally poured into two test tubes. 2–4 drops of Dragendoff's reagent were poured into one test tube, whereas 2–4 drops of Mayer's reagent were poured into the second test tube. The existence of alkaloids was determined with the presence of yellow precipitate in the mixture [20].

#### 2.2.3. Flavonoids and Flavones Test

Flavonoids and flavones were assessed by adding 4 ml of diluted sodium hydroxide (NaOH) into the 6 ml fermentation solution, which resulted in the appearance of yellow colour of the mixture. Then, 2 ml 5N hydrochloric acid (HCl) was added into the mixture, which made it colourless confirming the existence of flavonoids, while orange colour confirmed flavones [19].

#### 2.2.4. Phenols Test

Presence of phenols was determined according to Ahmed et al. [21]. 3 ml distilled water and 3-4 drops of 10% ferric chloride (FeCl_3_) were poured into 1.5 ml of the fermentation solution. The appearance of a bluish-green colour showed the existence of phenols in the mixture; on the other hand, addition of 4 ml of fermentation solution into 6 ml of 10% lead acetate formed white precipitation, confirming the presence of phenols.

#### 2.2.5. Steroids and Terpenoids Test

For the estimation of steroids and terpenoids, 1 g of fermentation was dissolved into 4 ml chloroform. The mixture was filtrated and then kept in an icebox. 4 ml of acetic acid and 4-5 drops of 100% sulphuric acid (H_2_SO_4_) were carefully poured into the mixture. The appearance of a pinkish-brown or pink ring confirmed the presence of terpenoids, whereas bluish-green or blue colour for steroids existence and the mixture of both colours indicated the existence of terpenoids and steroids [22].

#### 2.2.6. Saponins Test

For the estimation of saponins, 1 g of fermentation was added into 20 ml distilled water and shaken energetically. The development of 1 cm foam layer confirmed the existence of saponins [23].

#### 2.2.7. Tannins Test

Tannins existence was assessed by adding 2 ml distilled water and 2–4 drops of concentrated ferric chloride (FeCl_3_) into 1 ml of fermentation solution. Greenish-black or blue colour appearance demonstrated the existence of catecholic and gallic tannins, respectively [23].

### 2.3. Quantitative Chemical Analysis

#### 2.3.1. Determination of Total Flavonoids Content (TFC)

Total flavonoids content was assessed by the aluminium chloride colourimetric method using sodium hydroxide (NaOH) and aluminium trichloride (AlCl_3_). Briefly, 2 ml of fermentation solution was poured into 6 ml methanol, with further addition of 10 ml distilled water, 0.2 ml 1M potassium acetate (CH_3_COOK), and 0.4 ml 10% aluminium chloride (AlCl_3_) into the mixture. Then the subsequent solution was incubated for 30 min at room temperature in darkness. Absorbance was calculated at 420 nm in a 96-well ELISA plate using an “absorbance microplate reader” (SpectraMax 190, designed in USA, manufactured in China). Quercetin was used as the standard (1 mg/ml) solution in order to obtain the calibration curve using a series of concentrations, i.e., 0, 0.01, 0.02, 0.05, 0.10, 0.25, 0.50, and 1 mg/ml. Total flavonoid content was calculated from the calibration curve by using linear regression equation (*Y* = −0.004*x* + 0.053; *R*^2^ = 0.995). Consequences were shown as quercetin equivalent (QE) mg/g of fermentation. The experiment was performed in triplicate [24].

#### 2.3.2. Determination of Total Phenolic Content (TPC)

Total phenol content was calculated by using Folin–Ciocalteu reagent procedure with minor alterations. 2 ml fermentation solution was added to 4 ml of 2% sodium carbonate (Na_2_CO_3_) and 5 ml of 10% Folin–Ciocalteu reagent. The subsequent solution was incubated for 15 min at room temperature in darkness. Then, to quantify absorbance at 765 nm, the resulting mixture was poured into a 96-well ELISA plate. The standard curve was made by gallic acid (1 mg/ml). Outcomes were measured through the standard curve achieved by a series of gallic acid concentrations (0, 0.01, 0.02, 0.05, 0.10, 0.25, 0.50, and 1 mg/ml). The phenolic content was calculated from the calibration curve by using linear regression equation (*Y* = −0.005*x* + 0.053; *R*^2^ = 0.998). Consequences are determined as gallic acid equivalent (GAE) mg/g of compounds. The experiment was performed in triplicate [24].

#### 2.3.3. Determination of DPPH Radical Scavenging Activity

Antioxidant activity was calculated on the basis of the scavenging activities of DPPH radical (1,1-diphenyl-1-picrylhydrazyl). 0.5 ml of fermentation solution was added into freshly prepared 3.5 ml of DPPH methanolic solution (0.004 g/100 ml). The mixture was incubated for 30 min at room temperature in darkness and was poured into a 96-well ELISA plate to observe the absorbance at 517 nm. Percentage of inhibition of DPPH was measured by the reduction of absorbance by using the following equation. A less absorbance indicates more free radical scavenging potential or vice versa [25].

(1) $$\begin{array}{c} \text{Inhibition percent}\left(I\%\right)=\frac{A_{\text{blank}}-A_{\text{sample}}}{A_{\text{blank}}\;}\times 100, \end{array}$$

where *A*_blank_ is absorbance in the control and *A*_sample_ is absorbance in the sample.

### 2.4. Preparation of Sample for Gas Chromatography-Mass Spectrophotometry (GC-MS)

Fermentation was prepared for Gas chromatography-mass spectrometry as previously described in a procedure by Popova et al. [26] with minor modifications. 0.05 g of dry fermentation was dissolved into 5 ml of methanol HPLC grade. 200 *μ*L of the subsequent solution was mixed with 800 *μ*L of methanol for further dilution to make 1 ml and poured into a 1.5 ml centrifuge tube.

### 2.5. Gas Chromatography-Mass Spectrophotometry Analysis

Fermentation was analyzed by GC-MS using Agilent 6890-5973_N,_ USA. Gas chromatography (GC) equipped with an HP1 TG-5MS polydimethylsiloxane capillary column (30 m × 250 *μ*m × 0.25 *μ*m) interfaced with a Hewlett Packard 5973 mass selective detector. Parameters were fixed; 70°C was the initial temperature extended up to 220°C at an increase rate of 10°C per min. However, the inlet temperature was 250°C, split ratio 10 : 1, MS quadruple temperature 150°C, thermal Aux temperature 285°C, MS scan temperature 35–520 units, and ionization energy 70 eV; as a carrier gas, helium was used at a flow rate of 1.0 ml/min^−1^. Compounds were identified by construal on GC-MS mass spectrum by using the literature data or database at Wiley/NIST.98.1 [27]. The comparative yield of each compound was assessed based on raw data areas of GC with no response factor correction of FID (flame ionization detection).

### 2.6. Statistical Analysis

All data were statistically analyzed by using one-way ANOVA (analysis of variance) in Duncan's multiple range test (*p*=0.05). All statistical analyses weres performed by using IBM-SPSS software (version 25.0).

## 3. Results and Discussion

### 3.1. Qualitative Mycochemical Analysis

Tests for chemical screening showed the existence of bioactive compounds, i.e., glycosides, phenols, flavonoids, flavones, saponins, and tannins in the fermentation of Snef1216 (*P. chrysogenum*) (see Table 1). The estimation of compounds was denoted as maximum existence (++) due to more similarity; moderate existence (+) due to average or weak and (−) without any similarity or alteration of colour of the reaction mixture. The detection of glycosides was determined by the appearance of pink colour. Green or blue colour showed the existence of phenols in the mixture. The detection of flavones and flavonoids was confirmed by the alteration of yellow reaction mixture into orange or colorless, respectively. The existence of saponins in the mixture was determined by the formation of a foaming layer in the solution. The appearance of black or green colour confirmed the presence of catecholic tannins.

The results of qualitative tests for chemical screening showed a moderate presence of glycosides while alkaloids were absent in *P. chrysogenum*. Besides, phenols and flavonoids highly existed, whereas flavones were moderately present. Qualitative chemical screening of fermentation revealed that steroids and terpenoids were absent, while a moderate presence of saponins and catecholic tannins was recorded. However, gallic tannins were not recorded during chemical screening. The chemical analysis of the nutritious medium displayed only a moderate presence of phenols and flavonoids.

Antioxidants are the key substances that possess defensive nature against damage posed by the free radicals that become a cause of induced oxidative stress. Mycochemicals or secondary metabolites are occurring naturally in fungi which are an essential source of natural antioxidants that possess curative, protective, and defensive potential [28]. These mycochemicals can play a useful role in several mechanisms such as transforming hydroperoxides into nonradical species, scavenging oxygen, binding of metallic ions, and neutralizing singlet oxygen or absorbing ultraviolet radiation [29]. Essential mycochemicals contained in fungi have attained more attention due to their characteristics to inhibit disease by their antioxidant activity.

Mycochemical screening exposed the existence of chemical constituents, i.e., glycosides, alkaloids, phenols, flavonoids, terpenoids, flavones, steroids, tannins, and saponins. Terpenoids are used as anti-inflammatory and anticancer agents and contain leishmanicidal and trypanocidal activities [30]. Tannins exhibit antibacterial, antitumor, and antiviral activities [10]. Alkaloids display a wide range of activities such as antitumor, antihypertensive, antidepressant, anticholinergic, myorelaxant, anti-inflammatory, sympathomimetic, antitussigen, hypnoanalgesic, diuretic, antitumor, emetic, and antimicrobial [31].

Our results agree with Jakovljević et al. [10] that terpenoids, alkaloids, flavonoids, and phenols are present in *P. cyclopium* and *P. brevicompactum.* Mycochemical screening of *P. frequentans* showed the existence of chemical constituents, i.e., terpenoids, phenols, saponins, tannins, flavonoids, and alkaloids while steroids were absent [32]. *Aspergillus* sp. JPY1, *Phoma* sp., and *P. chrysogenum* displayed the existence of terpenes, tannins, sterols, and flavonoids [4]. This study demonstrated that the qualitative mycochemical screening of Snef1216 (*P. chrysogenum*) exhibited vital bioactive chemical constituents.

### 3.2. Quantitative Mycochemical Analysis

#### 3.2.1. Total Flavonoids Content (TFC)

The total amount of flavonoids contents was calculated through the standard curve using serial concentrations of quercetin, namely, 0, 0.01, 0.02, 0.05, 0.10, 0.25, 0.50, and 1 mg/ml. The total flavonoids contents of *P. chrysogenum* and nutritious medium are presented in Table 2. The flavonoids contents of *P. chrysogenum* and nutritious medium were 85.31 ± 1.23 and 1.43 ± 1.33 mg quercetin equivalent QE/g, respectively. The nutritious medium displayed fewer flavonoid contents as compared to fermentation strain Snef1216 (*P. chrysogenum*).

Flavonoids are the major secondary metabolites of fungi [33, 34]. They are polyphenolic compounds recognized because of their antioxidant properties and free-radical scavenging potential [35]. They act as potential therapeutic agents such as antiviral, antiherbivore, antipyretic, antiulcerogenic, antimutagenic, antitumor, antibacterial, antibiotic, antiprotozoal, anticarcinogenic, antinociceptive, and anti-inflammatory activities and antioxidants [36]. Bhardwaj et al. [32] described that flavonoids content was present in *P*. *frequentans* (17.48 mg/g), *Alternaria alternate* (17.41 mg/g), *Thielaviopsis basicola* (17.41 mg/g), and *Geotrichium albida* (17.41 mg/g). Nutritious medium displayed fewer flavonoid contents as compared to fermentation strain Snef1216 (*P. chrysogenum*). Mostly, the enrichment of antioxidants takes place during the fermentation because of the linkage between the breakdown of flavonoid compounds and microorganisms [37]. Additionally, fermentation prompts the structural breakdown of large and complex molecules which lead to the release or synthesis of various antioxidant compounds [38, 39]. Our study indicates that Snef1216 (*P. chrysogenum*) is an important source of flavonoid compounds.

#### 3.2.2. Total Phenolic Content (TPC)

The total amount of phenolic content was measured through a standard curve using serial concentrations of gallic acid (0, 0.01, 0.02, 0.05, 0.10, 0.25, 0.50, and 1 mg/ml). The total phenolic contents of *P. chrysogenum* and nutritious medium were presented (Table 2) as 135.77 ± 1.14 and 3.68 ± 0.96 mg gallic acid equivalent GAE/g, respectively. However, a nutritious medium showed very less phenolic content as compared to fermentation strain Snef1216 (*P. chrysogenum*). During fermentation, bioactive compounds may be produced through secondary metabolic pathways or released from the substrate by enzymes produced through microorganisms [40].

Phenolic compounds derived from fungi showed antioxidant properties by stopping the conversion of hydroperoxides into nonradicals or deactivating lipid free radicals [41]. The present study revealed that the high amount of phenolic content was recorded in fermentation of Snef1216 (*P. chrysogenum*). In comparison, a nutritious medium showed very few phenolic contents. However, our results were contradictory to Jakovljević et al. [6] who reported that *P. chrysogenum* had a lower content of total phenols (2.859 mg·GAE/g) than of *P. fumiculosum* (2.109 mg·GAE/g). The amount of total phenolic content in *P. chrysogenum* was 15.45 mg·GAE/g [42]. During the present study, the higher amount of phenolic content was recorded in the fermentation of *P. chrysogenum*. Similarly, earlier researchers have observed that fermentation can efficiently enhance the phenolic content of any tested materials [43–45]. Basically, these enhancements in total phenolic contents were due to microorganisms which involved in fermentation [46, 47]. Snef1216 (*P. chrysogenum*) may attain as an important source of phenolic compounds, contributing a better choice for the production of natural antioxidants.

#### 3.2.3. DPPH (1,1-Diphenyl-1-Picrylhydrazyl) Radical Scavenging Activity

DDPH (1,1-diphenyl-1-picrylhydrazyl) is a free and stable radical which displayed colour absorption at 517 nm. These radicals can be scavenged by the different types of antioxidant molecules because these radicals can easily donate their hydrogen molecules, and as a result, colour of the solution of DPPH (1,1-diphenyl-1-picrylhydrazyl) turns into light yellow initiating decrease in absorbance. DPPH radicals are widely used for the assessment of radical scavenging activities. Table 2 represents the DPPH scavenging activity of the fermentation strain Snef1216 (*P. chrysogenum*) and nutritious medium. DPPH inhibition (%) of Snef1216 (*P. chrysogenum*) was recorded as 63.86 ± 0.82. Moreover, the nutritious medium displayed no DPPH radical scavenging activity.

Outcomes of DPPH scavenger action studies showed that the percentage inhibition of DPPH was 51.34% in *P. fumiculosum* and 37.42% in *P. chrysogenum* [6], while during the present study, DPPH inhibition (%) of Snef1216 (*P. chrysogenum*) was recorded as 63.859 ± 0.823. It has been reported in the previous study that DPPH radical scavenging ability was significantly enhanced by fermentation [48]. Chomcheon et al. [49] demonstrated that *Aspergillus niger* exhibited notable antioxidant compounds during fermentation. Our finding indicates that Snef1216 (*P. chrysogenum*) may accomplish as a natural antioxidant agent.

### 3.3. Gas Chromatography-Mass Spectrometry Analysis

**Figure 1:**
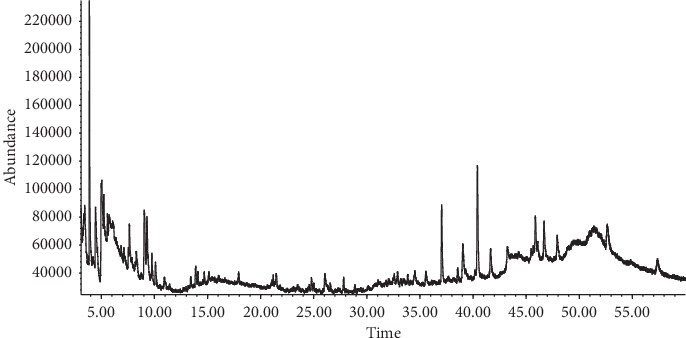
Biochemical composition of fermentation of Snef1216 (*P. chrysogenum*).

The existence of biologically active compounds in Snef1216 (*P. chrysogenum*) was screened through conducting GC-MS analysis. The principal compound, peak area (%), retention time (R.T), and molecular formula (M.F) with their molecular weight g/mol (M.W) are presented in Table 3. The results pertaining to GC-MS analysis of fermentation of Snef1216 showed the presence of ten compounds corresponding to 100% of total fermentation. However, benzoic acid, 2-methoxy-, methyl ester (23.51%), hexadecanoic acid, methyl ester (18.68%), undecane (11.94%), isopropyl myristate (11.66%), and oxime-, methoxy-phenyl (9.22%) were the major compounds while others were recorded in low quantity with the peak value ranged from 3.83–5.78%. The chromatogram obtained by GC-MS analysis detected different peaks of compounds (see Figure 1).

These biologically active compounds are accountable for antioxidant activities. Baert [50] reported that benzoic acid, 2-methoxy-, methyl ester, or ortho-anisic acid can be used as a nonsteroidal anti-inflammatory drugs. Oxime-, methoxy-phenyl, and hexadecanoic acid possess antioxidant and antimicrobial behaviour [51, 52]. The compounds cyclotrisiloxane, hexamethyl, and isopropyl myristate have the antioxidant potential [53, 54]. Undecane has a broad spectrum of antimicrobial activities [55]. Benzoic acid methyl ester displayed repellent potential towards insects such as *Bemisia tabaci* [56]. Chen et al. [57] also reported that methyl benzoate has insecticidal properties against pest insects and mites. Save et al. [58] reported that 1, 2-benzenedicarboxylic acid, butyl octyl ester, can be used in drug development for arthritis, microbial allergies, and cancer, whereas it can also be used as an antioxidant and antimicrobial agent [59]. 8-Octadecenoic acid methyl ester (E) showed antioxidant and antimicrobial activity and also effects of the serum lipid in women during lactation period [60, 61]. Merlin et al. [62] reported that heptadecanoic acid, 16-methyl, methyl ester possess anti-inflammatory, antimicrobial, and antioxidant properties. Almost all compounds contained in Snef1216 (*P. chrysogenum*) are important from the pharmacological and agricultural viewpoint.

## 4. Conclusion

Based on the outcome of the present study, it was concluded that fermentation strain Snef1216 (*P. chrysogenum*) comprised of a numerous secondary metabolites; flavonoids and phenols confer to free-radical scavenging activities. It could be an effective and safe source of natural antioxidant. Mycochemical screening revealed the existence of ten biologically active compounds responsible for the antioxidant activity of Snef1216 (*P. chrysogenum*). The present study provides the basis for the use of Snef1216 (*P. chrysogenum*) as natural antioxidant agent. However, further studies are desired to explore the role of mycochemicals accountable for the antioxidant activity of Snef1216 for its commercial use in the agricultural and pharmaceutical industry.

## Figures and Tables

**Table 1:** Qualitative analysis of mycochemicals in the fermentation strain Snef1216 (*P. chrysogenum*) and nutritious medium.

Mycochemical constituents	Snef1216 (*P. chrysogenum*)	Nutritious medium
Glycosides	+	—
Alkaloids	—	—
Flavonoids	++	+
Flavones	+	+
Phenols	++	—
Terpenoids	—	—
Steroids	—	—
Saponins	+	—
Catecholic tannins	+	—
Gallic tannins	—	—

**Table 2:** Total flavonoids contents, total phenolic contents, and DPPH radical scavenging activity of fermentation of Snef1216 (*P. chrysogenum*) and nutritious medium.

Quantitative analysis	Total flavonoids contents (mg·QE/g)	Total phenolic contents (mg·GAE/g)	DPPH inhibition (%)
Snef1216 nutritious medium	85.31 ± 1.23	135.77 ± 1.14	63.86 ± 0.82
	1.43 ± 1.33	3.68 ± 0.96	0 ± 0

Statistical summary	S.S = 8637.65	S.S = 18848.02	S.S = 2885.37
	D*f* = 1, *p*=0.000	D*f* = 1, *p*=0.000	D*f* = 1, *p*=0.000
	M.S = 8637.65	M.S = 18848.02	M.S = 2885.37
	*F* = 5273.10	*F* = 16948.30	*F* = 8527.20

Values are presented in the table as mean ± standard deviation. S.S, sum of square; M.S, mean square; D*f*, degree of freedom; *F*, *F* value; *p*, significant value.

**Table 3:** Biochemical composition of fermentation of Snef1216 (*P. chrysogenum*).

P. no.	R.T	Compounds	Area (%)	M.F	M.W (gmol^−1^)
1	3.876	Benzoic acid, 2-methoxy-, methyl ester	23.51	C_6_H_10_O_3_	166.17
2	4.448	Oxime-, methoxy-phenyl	9.22	C_8_H_9_NO_2_	151.16
3	7.643	Cyclotrisiloxane, hexamethyl	3.83	C_6_H_18_O_3_Si_3_	222.46
4	9.043	Undecane	11.94	C_11_H_24_	156.31
5	9.298	Benzoic acid, methyl ester	5.78	C_6_H_5_COOCH_3_	136.15
6	37.043	Isopropyl myristate	11.66	C_17_H_34_O_2_	270.5
7	40.415	Hexadecanoic acid, methyl ester	18.68	C_17_H_34_O_2_	270.45
8	41.654	1, 2-Benzenedicarboxylic acid, butyl octyl ester	4.49	C_20_H_30_O_4_	333.45
9	45.865	8-Octadecenoic acid, methyl ester (E)	5.45	C_19_H_36_O_2_	296.5
10	46.687	Heptadecanoic acid, 16-methyl-, methyl ester	5.44	C_19_H_38_O_2_	298.50

P. no., peak number; R.T, retention time; M.F, molecular formula; M.W, molecular weight.

## Data Availability

All the data supporting the current findings reported in this manuscript are available within the manuscript and not attached as any supplementary file.
